# Comparison of Two Different OCT Systems: Retina Layer Segmentation and Impact on Structure-Function Analysis in Glaucoma

**DOI:** 10.1155/2016/8307639

**Published:** 2016-02-04

**Authors:** Livia M. Brandao, Anna A. Ledolter, Andreas Schötzau, Anja M. Palmowski-Wolfe

**Affiliations:** ^1^Department of Ophthalmology, University of Basel, 4031 Basel, Switzerland; ^2^Department of Ophthalmology, Medical University of Vienna, 1090 Vienna, Austria

## Abstract

*Purpose*. To compare two different spectral-domain optical coherence tomography (OCT) systems in regard to full macular thickness (MT) and ganglion cell layer-inner plexiform layer (GCIPL) measures and in regard to structure-function correlation when compared to standard automated perimetry (SAP).* Methods*. Seventeen primary open angle glaucoma patients and 16 controls (one eye per subject) were enrolled. MT and GCIPL thicknesses were measured by Cirrus and Spectralis OCTs. Octopus Perimeter 101 (G2 protocol) reports sensitivity in mean defect (dB). Differences between measurements were assessed with Student's *t*-test and Bland Altman. Diagnostic performance was also compared between each parameter calculating the areas under the operator receiver (ROC). Linear models were used to investigate structure-function association between OCT and SAP.* Results*. Disagreement between OCTs in both MT and GCIPL values was significant. Spectralis values were thicker than Cirrus. Average difference between OCTs was 21.64 *μ*m (SD 4.5) for MT and 9.8 *μ*m (SD 5.4) for GCIPL (*p* < 0.001). Patients differed significantly from controls in both OCTs, in both measurements. MT and GCIPL were negatively associated with MD (*p* < 0.001).* Conclusions*. Although OCT values were not interchangeable, both machines differentiated patients from controls with statistical significance. Structure-function analysis results were comparable, when either OCT was compared to SAP.

## 1. Introduction

Glaucoma remains the third or fourth most common cause of blindness in different regions around the globe. Since this is a treatable disease, early diagnosis and adequate follow-up are gaining importance. Also, direct and indirect economic burdens tend to increase for governments with patients extended lifetime expectancy and complexity of disease stage [1].

So far standard automated perimetry (SAP) is still considered the “gold standard” method for function analysis even though defects are only detectable after substantial cell loss [2]. Since its introduction, optical coherence tomography (OCT) has turned into a fundamental tool in the evaluation of a variety of different retinal diseases, in particular primary open angle glaucoma (POAG). Thinning of the peripapillary retinal nerve fiber layer (RNFL) and full macular thickness (MT) have been largely used in POAG evaluation [3, 4]. In addition, recent improvements in OCT technology (i.e., Spectral OCT and software analysis) not only increased image resolution but also allowed customized analysis of the individual retinal layers. Glaucoma damage is primarily related to the ganglion cells [5]. Thus OCT retinal layer segmentation allows us to directly analyze the ganglion cell layer separately and thereby look directly at the site of damage. Indeed, ganglion cell-inner plexiform layer (GCIPL) segmentation can identify changes and correctly diagnose glaucoma with a similar sensitivity as the RNFL or optic nerve head (ONH) parameters [6–8]. Thus segmentation may allow a more sensitive structure-function correlation in different stages of disease. Although the increased number of OCT manufacturing companies may contribute positively to price competition as well as hardware and software improvements, it also leads to variability in measurements and analysis methodology.

In this study, we determined if the brand of the spectral-domain OCT used and their respective segmentation programs influence structure-function analysis differently. To the best of our knowledge, this is the first study to compare two different OCT systems and their respective macula layer segmentation software and associate them with SAP in structure-function analysis.

## 2. Materials and Methods

The study protocol was approved by the Ethics Committee of the University of Basel, and informed consent was obtained from all participants before the examination. All procedures followed the tenets of the Declaration of Helsinki.

Patients with established glaucoma diagnosis and controlled IOP were recruited from the glaucoma ambulatory. The inclusion criteria included a visual acuity of 0.8 or better, and a refractive error between ±6 diopters of hyperopia or myopia. All glaucoma patients had a cup-to-disc ratio of at least 0.5 and a localized thinning of the neuroretinal rim on OCT (Cirrus) corresponding to the fundus examination. The OCT thinning should have at least one red sector or two yellow sectors on the thickness map (less than 1% and 5% of the normal population, resp., as by Cirrus software analysis). Preperimetric glaucoma was defined by the presence of optic nerve abnormalities consistent with glaucoma and a normal visual field as tested with SAP. Other glaucoma patients had to present a reproducible glaucomatous visual field defect on at least three examinations with a mean defect (MD) higher than 2.0 dB and/or a squared root of loss variance (sLV) over 2.5 dB. Individuals with previous ocular surgery, systemic diseases, or regular use of medications that could influence the eye (e.g., antidepressant, chloroquine) were excluded from the study.

The right eye was included in the study, if it did not fulfill any exclusion criteria. All OCT images were performed in the same day. When not possible, visual field examination was performed at a maximum interval of 7 months from OCT examination. Subjects underwent OCT imaging and SAP testing as described below. Data from the corresponding areas of the central 10° were analyzed.

**Figure 1:**
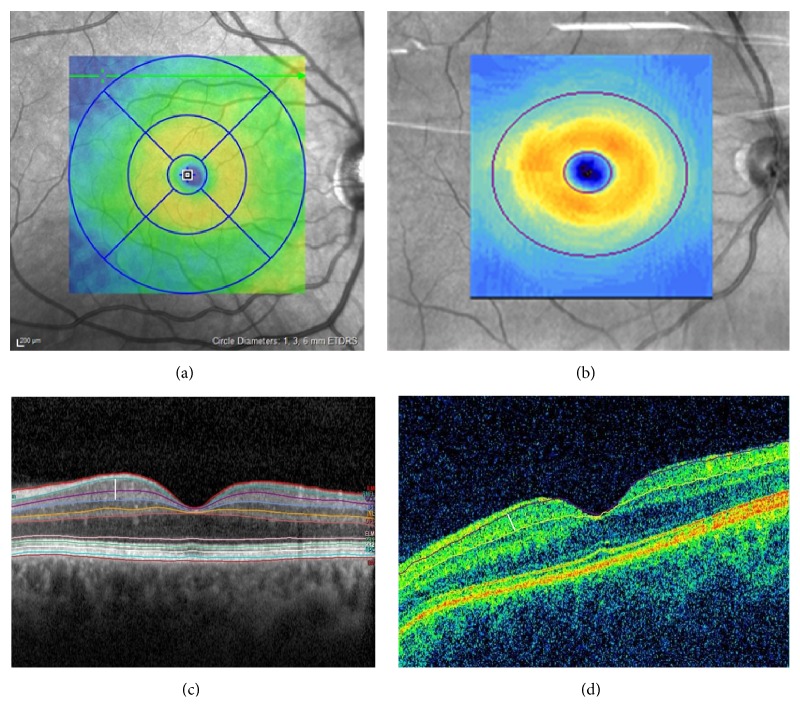
Top: (a) example from Early Treatment Diabetic Retinopathy Study (ETDRS) grid taken from Spectralis printout, used to calculate MT in both OCTs: scan area of 6 × 6 mm, divided into three concentric circles with 1 mm, 3 mm, and 6 mm diameter, respectively. Here, we used values from the 1 and 3 mm circles of the grid. (b) Area used in Cirrus to calculate GCIPL thickness corresponding to an elliptical annulus with a 2.0 mm vertical and 2.4 mm horizontal radius, excluding a central elliptical area (0.5 mm vertical and 0.6 mm horizontal radius) that corresponded to the foveola; in Spectralis GCIPL thickness was calculated with values in the 3 mm circle from ETDRS grid, excluding the 1 mm diameter circle. Bottom: OCT images using layer segmentation software from each instrument: (c) Spectralis, (d) Cirrus (same patient). While Cirrus software calculates only ganglion cell-inner plexiform layer (GCIPL) and excludes retina nerve fiver layer (RNFL), Spectralis software segments all retina layers and values from ganglion cell and inner plexiform layers were added manually. The white bar represents limits for thickness measurement of ganglion cell-inner plexiform layer in Spectralis (left) and Cirrus (right).

Technical details from the two different commercially available OCT instruments used are displayed in Table 1. The pupil of the study eye was dilated with a solution of tropicamide 0.5% and phenylephrine 1% (Spital-Pharmazie USB, Switzerland) before examination. OCT images were obtained in Cirrus using the fast macular cube protocol 512 × 218 (128 horizontal scan lines each composed of 512 A-scans, Cirrus SD-OCT, Carl Zeiss, USA), and the fast volume scan in Spectralis HRA + OCT (25 section scans and 26 ART frames, Heidelberg Engineering, Inc., Heidelberg, Germany). The same specialist executed all OCT images. Both instruments have a scan area of 6 × 6 mm and macular retinal thickness is calculated in microns in an area correspondent to the Early Treatment Diabetic Retinopathy Study (ETDRS) grid. The MT values used in this study corresponded to the 1 and 3 mm circles of the ETDRS grid. GCIPL thickness is calculated by Cirrus software in the area of an elliptical annulus with a 2.0 mm vertical and 2.4 mm horizontal radius, excluding a central elliptical area (0.5 mm vertical and 0.6 mm horizontal radius) that corresponded to the foveola. According to studies of human retina, the highest density of ganglion cells occurs in this area [9]. As Spectralis software (version 6.0.3) uses the ETDRS grid also for GCIPL thickness calculation, values in the 3 mm circle were averaged, excluding the 1 mm circle, and compared to Cirrus (Figure 1, top).

Also the segmentation software from each OCT calculates layer thickness differently. Cirrus software excluded the macular RNFL layer from the GCIPL analysis while Spectralis software calculated each retina layer separately (Figure 1, bottom). Therefore, in Spectralis, only the layers of interest in this study (ganglion cell and inner plexiform layers) were added in a separate Microsoft Excel spreadsheet. The exclusion of RNFL in Cirrus was based on the histologic observation that the macular GCIPL layer presents less variation than the RNFL among normal individuals [6]. Differences between the OCTs are that while both allow for manual corrections of the macular thickness boundaries, only Spectralis allows for manual correction of possible errors in GCIPL segmentation. Cirrus, but not Spectralis, separately analyses the minimum value of GCIPL thickness (mGCIPL) measured within the areas analyzed. Thus parameters included in this analysis were averaged MT and the GCIPL from both OCTs, and in addition, their average after manual correction (cMT and cGCIPL) in Spectralis and the mGCIPL value given in Cirrus. All images included in this study had signal strength over 7 for Cirrus and a quality score over 25 for Spectralis (limits considered as good/acceptable image quality for analysis, according to each instrument's manual).

Standard automated perimetry was performed using an Octopus Perimeter (Octopus 101, G2 Program, Haag-Streit AG, Switzerland). Total field MD (mean defect) values in dB were included in the analysis. All SAP exams used in this study were inside reliability parameters (fixation loss < 33%, false-positive and false-negative rates < 25%).

## 3. Statistical Analysis

Bland-Altman analysis was used to compare OCT results. Differences between measurements were compared using the paired *t*-test. Differentiation between glaucoma and controls within each measurement was assessed with a *t*-test and *p* values posteriorly adjusted with FDR (false discovery rate). The predictive diagnostic performance for each parameter, that is, the ability to differentiate glaucoma from control, was assessed calculating the receiver operating characteristics (ROC) curves and the respective area under the ROC curve (AUC). A perfect predictive performance is represented by an AUC of 1.0 which means that this parameter can differentiate glaucoma from control with 100% sensitivity and specificity, while an AUC value of 0.5 means a prediction mostly influenced by chance. AUCs from different OCTs were compared with the DeLong test.

For prediction of structure-function relationship, linear models were performed. All calculations were adjusted to age. As OCT values are linear values but SAP are reported in dB, a logarithmic value, OCT values were transformed to logarithmic scale for better comparisons with SAP. Results were expressed as the regressive slope coefficients (on log-scale) with corresponding standard errors and *p* values.

Statistical analyses were performed using SPSS (IBM SPSS Statistics, version 22), and the statistical package R [10] (version 3.0.2). In this study, all *p* values <0.05 were considered as significant.

## 4. Results

A total of 33 eyes were included: 17 POAG and 16 controls. Complete demographic details are presented in Table 2. The mean age was 59.5 years (SD ± 13.9) for the glaucoma group and 49.2 years (SD ± 7.0) for the controls (*p* = 0.013). Median MD was 2.2 (range: −0.4–17.0) dB in POAG (including PPG) and −0.2 (range: −3.8–2.0) dB in controls (*p* = 0.024).

### 4.1. Macular and GCIPL Thickness in OCT

**Figure 2:**
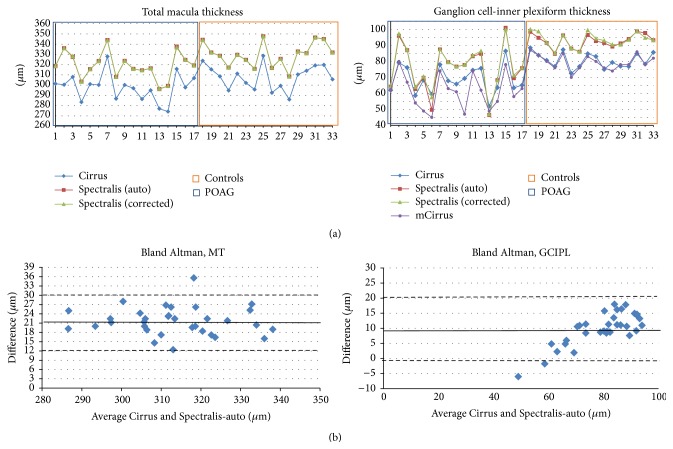
(a) This plot shows the measurements from both OCTs per patient: (left) total macula thickness; (right) ganglion cell-inner plexiform layer thickness. Comparison between these measurements allows visual appreciation of the difference per patient. (b) Respective Bland-Altman graphic representation comparing the difference between Cirrus and Spectralis MT (right) and GCIPL (left) to their mean. Auto: values using automatic segmentation in Spectralis; corrected: values after manual correction of retinal layer segmentation in Spectralis; mGCIPL: minimum GCIPL value calculated by Cirrus software. POAG: primary open angle glaucoma group; controls: control group.

Table 3 shows averages from MT and GCIPL in both OCTs in the POAG and control groups. Spectralis and Cirrus showed a significant difference between patients and controls in both MT (*p* = 0.018, *p* = 0.028, resp.) and GCIPL (*p* < 0.001, both). Manual correction of the software segmentation parameters in Spectralis produced a significant difference between measurements in GCIPL (*p* < 0.05) but not in MT (*p* = 0.715). A total of 2 controls (12.5%) and 7 patients (41.1%) needed posterior manual correction of retina thickness segmentation. No subject needed macula thickness segmentation correction in Cirrus analysis. Differences between OCT measurements per patient are shown in Figure 2(a).

Bland-Altman analysis showed disagreement between OCTs in MT and GCIPL values (Figure 2(b)). On average, measurements with Spectralis were thicker than with Cirrus. For MT the difference was 21.64 *μ*m (SD ± 4.5) before and 21.65 *μ*m (SD ± 4.5) after manual correction (*p* < 0.001). For average GCIPL thickness the difference was 9.8 *μ*m (SD ± 5.4) before and 10.0 *μ*m (SD ± 5.3) after manual correction (*p* < 0.001). With higher values measurements obtained with Spectralis tended to differ more from those measured with Cirrus. This difference increased when we compared Spectralis averages before (14.1 *μ*m, SD ± 5.9) and after correction (14.4 *μ*m, SD ± 5.8) with mGCIPL (*p* < 0.001).

**Figure 3:**
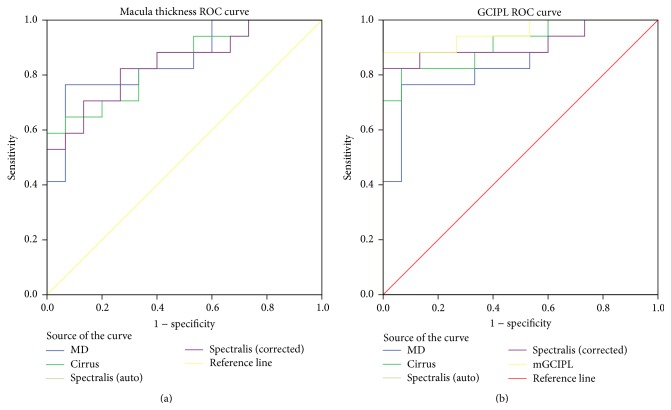
Age-adjusted ROC curves from Cirrus and Spectralis: (a) total macula thickness, (b) GCIPL. Auto: values using automatic segmentation in Spectralis; corrected: values after manual correction of retinal layer segmentation in Spectralis; mGCIPL: minimum GCIPL value calculated by Cirrus software; MD: mean defect.

There was no significant difference between the age-adjusted AUCs from MT in Cirrus (0.798) and Spectralis, before (0.801) and after (0.805) manual correction. This was also observed between OCTs for GCIPL measurements: 0.879 in Cirrus and 0.886 before and 0.886 after correction in Spectralis. Minimum GCIPL value in Cirrus had an AUC of 0.930 (Table 3; Figure 3).

### 4.2. Structure-Function Relationship

**Figure 4:**
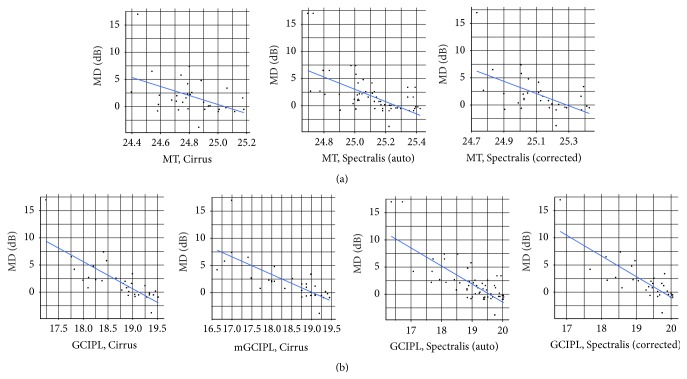
Scattered plots showing the structure-function relationship were obtained using linear models analysis. Age adjustment was applied in all calculations: top row from left to right: MD versus macular thickness from Cirrus, Spectralis (automatic values), and Spectralis (manually corrected values). Bottom row from left to right: MD versus ganglion cell-inner plexiform layer thickness from Cirrus (average and minimum value), Spectralis (automatic values), and Spectralis (manually corrected values). MD: mean defect.

The association between SAP and OCT was assessed using a linear model. MT and GCIPL had a negative significant association with MD (*p* < 0.001), in both POAG (*p* < 0.001) and controls (*p* < 0.001) for Cirrus and Spectralis (Table 4, Figure 4).

## 5. Discussion

The aim of this study was to compare thickness measurements between two commercially available OCTs using their respective segmentation programs and assess if the brand of spectral-domain OCT used might influence structure function analysis differently in glaucoma. Using two different spectral-domain OCTs, Cirrus and Spectralis, we observed that there is a significant difference in full macula and GCIPL thickness measurements between machines. Therefore measurements are not interchangeable.

Nevertheless, when assessing structure function relationship individually, all measurements from both machines demonstrated a statistically significant relationship with function measured by standard automated perimetry. Further, age-adjusted AUCs demonstrated that all measurements had a similar predictive performance and could correctly differentiate patients from controls.

A literature review in PubMed using specific terms (optical coherence tomography, glaucoma, ganglion cell, macula, thickness, and segmentation software) did not reveal any other study which we could directly compare to this one. While we compared the entire area within the central 3 mm diameters, most studies either refer to the central 1 mm area [11–16] or compare the areas within the ETDRS sectors calculated by the respective OCTs (i.e., the superior, inferior, temporal, and nasal sectors) [11, 16]. Here we showed that all thickness values from Spectralis were consistently higher than in Cirrus. This is in accordance with a study from Mylonas et al. [11], where Spectralis macula thickness also showed the highest values, in the central retinal thickness (CRT, 1 mm diameter) and individual sectors of the 3 mm area, in comparison to other OCTs (including Cirrus). Though the study was conducted in neovascular age-related macular degeneration patients (28 individuals), its control group (10 individuals) showed the same pattern. Other studies found the same difference between Cirrus and Spectralis in CRT [12, 14, 15].

There are numerous studies applying GCIPL thickness in glaucoma [6–8, 16–21]. The GCIPL average in early glaucoma patients from these studies (69.7 *μ*m) is comparable to our study average (68.5 *μ*m). A comparison of layer segmentation reproducibility was conducted at the IOWA University using Cirrus and their own segmentation software [22]. Here, the overall average from Cirrus GCIPL was reported as 70.0 *μ*m (SD ± 11.4) in glaucoma, which also did not differ much from the patient group average in our study (Cirrus). Recently, Martinez-de-la-Casa et al. demonstrated that, using Spectralis layer segmentation software, macular RNFL thickness was the only parameter to differentiate healthy subjects from glaucoma suspects [21]. We have not found a study comparing Spectralis and Cirrus segmentation software, most probably because Spectralis software was made commercially available only recently.

The clear difference in macula thickness between OCTs could be explained analyzing the specific retina boundaries established by each manufacturer. While the inner boundary is always the vitreoretinal interface, the outer retinal boundary varies between manufacturers. For Cirrus the outer boundary corresponds to the level of the interdigitations of the external layers of the photoreceptors in the retinal pigment epithelium (Verhoeff's membrane), while in Spectralis it is at Bruch membrane [11, 13, 15]. Nevertheless, the establishment of different boundaries for total macula thickness calculation cannot explain the significant difference between GCIPL thicknesses we found in this study. Different image resolution, intrinsic reflectance, and analysis algorithms within each software may influence this calculation. We also cannot exclude an influence from the different areas analyzed, that is, ellipsoid in Cirrus versus the annular in Spectralis. However both areas differ only slightly and include the highest density area for ganglion cells.

While total macula thickness boundaries can be manually corrected in both Cirrus and Spectralis, individual layer segmentation correction is possible only in Spectralis. We did not observe a significant difference in total macula averages before and after manual correction of inner and outer retinal boundaries in Spectralis. This could be explained by the observation that, specifically for the internal limiting membrane and Bruch's membrane, delineation errors occurred mostly in the extreme periphery of the image slice. However, for the GCIPL segmentation errors also occur within the 3 mm ring averages. Thus GCIPL layer segmentation corrections made in Spectralis resulted in significantly different values, while values remained significantly higher than with Cirrus. In addition, the same difference in thickness measure will impact less on the thicker total macula thickness but more on the thinner GCIPL thickness. This probably explains the significant difference we found.

A significant correlation between function (global MD) and morphology (MT [23, 24] or GCIPL [20, 25, 26]) has been demonstrated previously. In agreement, despite the significant difference between Cirrus and Spectralis measurements, both OCTs demonstrated a significant positive association with global MD. We are not aware of studies directly comparing the diagnostic performance between MT and GCIPL in glaucoma. When compared to RNFL, MT had an inferior diagnostic performance in Cirrus [27] and in Stratus [3, 23]. In our study, Spectralis MT showed similar diagnostic performance to Cirrus. However both were outperformed by GCIPL measurements, especially mGCIPL. Even though we found no other studies directly comparing AUC between MT and GCIPL, the AUC values found in this study are in accordance with findings from other studies using MT [27] and GCIPL [7, 8, 17, 20, 28].

Though we found a significant difference between OCT measurements, the small population analyzed here may limit our findings. Also, stage of disease might influence results given that the relationship between structural and functional damage is still not completely understood. Including more patients and later stages of disease glaucoma could give us additional information. In addition, knowing that age may influence our results, we adjusted all calculations for age.

Difference in gender distribution is also a concern: Cohn et al. did not observe a significant difference between males and females when comparing total dB from SAP [29]. However according to Ooto et al. total macular thickness is about 7.5 *μ*m thicker in men than in women [30]. While age and sex differences should be considered when performing disease diagnostics, this is not the main scope of this study as we aimed to analyze measurements from two different OCTs within the same subject.

In contrast to Cirrus, Spectralis software does not yet include a normative databank. Once this is incorporated, comparison between deviation maps from these OCTs could contribute to understanding differences between calculations. Finally, the Spectralis segmentation software used here is a beta version. A definite version, without many changes, was recently made commercially available by the company.

In conclusion, the significant difference between measurements from Cirrus and Spectralis OCTs does not allow free interchange of machines, for instance, in the follow-up of patients. In a clinical setting, clinicians must be aware that once you change the machine and software analysis, a new baseline for the patient is needed. Nevertheless both machines showed similar capability of diagnostic performance in early glaucoma and also in their correlation to functional changes such as standard automated perimetry.

## Figures and Tables

**Table 1:** Technical characteristics from Cirrus and Spectralis OCT and acquisition protocol details of each device.

	Cirrus*™*	Spectralis*™*
Axial resolution	5 *µ*m	4 *µ*m
Scan speed (scan/sec)	27.000	40.000
Scan pattern	512 × 128	512 × 49
Scan area	6 × 6 mm	6 × 6 mm
Acquisition time	2.5 sec	5.0 sec
Software version	6.5.0	6.0.3

**Table 2:** 

Group	POAG (*n* = 17)	Controls (*n* = 16)	*p* value^*∗*^
Age (yrs) (mean ± SD)	59.5 ± 13.9	49.2 ± 7.0	*p* = 0.029
Gender (M/F)	12/5	4/12	
BVCA (decimal) (median, range)	1.0 (0.9–1.25)	1.2 (0.9–1.25)	*p* = 0.030
Refraction (mean ± SD)			
Diopters	−0.38 ± 2.0	−0.61 ± 2.1	*p* > 0.05
Cylinder	0.65 ± 0.72	0.72 ± 0.65	*p* > 0.05
MD (dB) (median, range)	2.2 (−0.4–17.0)	−0.2 (−3.8–2.0)	*p* = 0.024
RNFL (*µ*m) (mean ± SD)	69.4 ± 11.1	90.0 ± 10.6	*p* < 0.001
IOP (mmHg) (mean ± SD)	12.8 ± 1.7	13.3 ± 2.7	*p* = 0.811
CDR (median, range)	0.8 (0.5–0.9)	0.3 (0.2–0.4)	*p* < 0.0001

POAG: primary open angle glaucoma, SD: standard deviation, BVCA: best corrected visual acuity (decimal), MD: mean defect (dB), RNFL: retinal nerve fiber layer average thickness, IOP: intraocular pressure under medication (mmHg), CDR: cup-to-disc ratio. ^*∗*^ *p* values were obtained with *t*-test and posterior adjustment with FDR (false discovery rate).

**Table 3:** Total macula and ganglion cell layer mean thickness, in micrometers (*µ*m), among groups and *p* values in the central 10°.

	POAG	Controls	*p* value^*∗*^	AUC
MT (mean ± SD)				
Cirrus				
MT	297.6 ± 13.5	307.9 ± 12.1	**0.036**	0.789
Spectralis				
MT	319.0 ± 13.0	329.8 ± 11.7	**0.027**	0.801
cMT	319.0 ± 12.9	329.8 ± 11.7	**0.027**	0.805

GCIPL (mean ± SD)				
Cirrus				
GCIPL	68.5 ± 8.7	80.7 ± 4.7	**<0.01**	0.879
mGCIPL	61.2 ± 10.8	79.5 ± 4.7	**<0.01**	0.930
Spectralis				
GCIPL	75.7 ± 13.8	93.3 ± 4.6	**<0.01**	0.886
cGCIPL	76.1 ± 13.7	93.4 ± 4.6	**<0.01**	0.886

MT: full macular thickness, GCIPL: ganglion cell-inner plexiform layer thickness, cMT and cGCIPL: average values after manual correction of layer segmentation in Spectralis OCT, mGCIPL: minimum GCIPL value calculated by Cirrus software. AUC: area under the ROC curve. SD: standard deviation. ^*∗*^ *p* values were obtained with paired *t*-test and posterior adjustment with FDR (false discovery rate).

**Table 4:** Structure-function relationship expressed as regression coefficients and corresponding *p* values.

Independent variable	Dependent variable	Regressive slope	Std. error	*p* value
Cirrus MT	MD	−7.459	1.552	<0.001
Spectralis MT	MD	−9.590	1.656	<0.001
Spectralis cMT	MD	−9.559	1.671	<0.001
Cirrus GCIPL	MD	−4.623	0.445	<0.001
Cirrus mGCIPL	MD	−3.206	0.374	<0.001
Spectralis GCIPL	MD	−3.053	0.336	<0.001
Spectralis cGCIPL	MD	−3.548	0.315	<0.001

OCT values were transformed to logarithmic scale. MT: full macular thickness, GCIPL: ganglion cell-inner plexiform layer, cMT and cGCIPL: average values after manual correction of layer segmentation in Spectralis OCT, mGCIPL: minimum GCIPL value calculated by Cirrus software, MD: mean defect.
